# Limitations of Semi-Automated Immunomagnetic Separation of HLA-G-Positive Trophoblasts from Papanicolaou Smears for Prenatal Genetic Diagnostics

**DOI:** 10.3390/diagnostics15030386

**Published:** 2025-02-06

**Authors:** Eddy N. de Boer, Nicole Corsten-Janssen, Elles Wierenga, Theo Bijma, Jurjen T. Knapper, Gerard J. te Meerman, Gwendolyn T. R. Manten, Nine V. A. M. Knoers, Katelijne Bouman, Leonie K. Duin, Cleo C. van Diemen

**Affiliations:** 1 Department of Genetics, University Medical Center Groningen, University of Groningen, 9700 RB Groningen, The Netherlands; n.corsten@umcg.nl (N.C.-J.); e.wierenga@umcg.nl (E.W.); j.t.knapper@umcg.nl (J.T.K.); g.j.te.meerman@gmail.com (G.J.t.M.); v.v.a.m.knoers@umcg.nl (N.V.A.M.K.); k.bouman@umcg.nl (K.B.); c.c.van.diemen@umcg.nl (C.C.v.D.); 2 Flow Cytometry Unit, Pathology & Medical Biology, University Medical Center Groningen, University of Groningen, 9700 RB Groningen, The Netherlands; 3 Department of Obstetrics, Isala Clinic, 8025 AB Zwolle, The Netherlands; g.t.r.manten@isala.nl; 4 Department of Obstetrics, University Medical Center Groningen, University of Groningen, 9700 RB Groningen, The Netherlands; l.k.duin@umcg.nl

**Keywords:** prenatal genetic diagnostics, single-gene defects, fetal trophoblasts, Papanicolaou smear, semi-automated immunomagnetic cell sorting

## Abstract

**Background:** In prenatal genetic diagnostics, the detection of single-gene defects relies on chorionic villus sampling (CVS) and amniocentesis, which carry a miscarriage risk of 0.2–0.3%. To mitigate this risk, fetal trophoblasts have been isolated from a Papanicolaou smear using Trophoblast Retrieval and Isolation from the Cervix (TRIC). However, this method is labor-intensive and has been shown to be challenging to implement in clinical practice. Here, we describe our experiences in using semi-automated immunomagnetic cell sorting for isolating trophoblasts from clinically obtained Papanicolaou smears during ongoing pregnancies. **Methods:** Using HLA-G-positive Jeg-3 and HLA-G-negative HeLa cell lines in 10%, 1%, and 0.1% dilutions, we tested and optimized the isolation of HLA-G-positive cells using FACS and semi-automated immunomagnetic cell sorting. We used the latter technique for isolation of HLA-G-positive cells from Papanicolaou smears collected from 26 pregnant women, gestational age between 6 and 20 weeks, who underwent CVS. **Results:** In four independent dilution series, the mean percentages of Jeg-3 cells went from 7.1% to 53.5%, 0.9% to 32.6%, and 0.4% to 2.6% (7.5, 36, and 6.5-fold enrichment, respectively) using immunomagnetic cell sorting. After sorting of the Papanicolaou smears, HLA-G-positive cells were moderately increased in the positive (14.61 vs. 11.63%) and decreased in the negative fraction (7.87 vs. 11.63%) compared to baseline pre-sorting. However, we could not identify fetal cells using XY-chromosomal FISH in a male sample. **Conclusions:** Our study supports previous findings that careful sampling of fetal cells from Papanicolaou smears in a clinical context poses significant challenges to cell retrieval.

## 1. Introduction

An estimated 3% of children conceived in the Netherlands have a birth defect of genetic origin [1], translating to approximately 5000 cases per year. These birth defects include single-gene defects, with more than 7000 described worldwide, structural variations, and chromosomal abnormalities, with Down syndrome (trisomy 21) being the most common [1].

Non-invasive prenatal testing (NIPT) using cell-free fetal DNA (cffDNA) from the pregnant woman’s blood is currently the primary choice for screening for trisomy 13, 18, and 21 worldwide [2,3]. NIPT can also be expanded to detect smaller copy number variations (CNVs). However, published studies have shown a relatively low predictive value of 40–50%, with no significant difference between CNVs < 10 Mb and those ≥10 Mb [4] and clear differences in the performance per chromosome [5]. The main cause for this is the limited coverage of the fetal genome in NIPT.

Prenatal testing for single-gene defects such as structural variants (SV), CNVs, and single-nucleotide variants (SNVs) cannot be performed using NIPT and is currently performed on fetal DNA samples obtained through chorionic villus sampling (CVS) or amniocentesis. These are, in contrast to NIPT, both invasive procedures, with a 0.2–0.3% miscarriage risk [6,7], which may deter women from choosing genetic testing. Several efforts have therefore been made to develop reliable, non-invasive prenatal tests for single-gene defects. One option is whole-exome sequencing (WES) on cffDNA from maternal blood. However, the cffDNA fetal fraction in maternal blood falls between 4 and 50% (mean 13.4%) between 11 and 20 weeks of gestation [8], and cffDNA is nonrandomly fragmented [9]. Because of this, most of the sequencing data generated will be from the mother. This makes the detection of single-gene defects in the fetal fraction very expensive because it requires ultra-high coverage cffDNA WES (4548× mean) and the WES of both parents [10]. To overcome this, other sources of fetal DNA, ideally intact fetal cells, need to be used [11,12].

Fetal extravillous trophoblasts in maternal blood have been shown to be suitable for targeted cell-based NIPT, but this requires whole-genome amplification, which leads to bias as well as a single origin for many sequenced fragments due to the low numbers of trophoblasts (11.8 per 30 mL at gestational weeks 12–13) [13,14]. This low number of cells limits extensive testing.

Previous studies have shown that fetal trophoblasts can also be isolated from a Papanicolaou (Pap) smear as early as 5 weeks of gestation [15]. The Trophoblast Retrieval and Isolation from the Cervix (TRIC) technique makes use of the fact that intact fetal cells with a trophoblast-like phenotype are naturally shed from the conceptus into the reproductive tract. Trophoblast cells express the fetal cell marker HLA-G, which can be used to isolate trophoblasts from HLA-G-negative maternal cervical and epithelial cells. Using TRIC, the fetal cell percentage was enriched from 0.0005% to 92.2 ± 6.5%, obtaining DNA from 700 fetal cells [16]. This is sufficient material for prenatal genetic testing of chromosomal abnormalities and single-gene defects [16], making this non-invasive method a candidate replacement for chorionic villus sampling and amniocentesis in the future. However, the published TRIC protocol has proven difficult to replicate [17]. It also has a low-throughput and is labor-intensive, making it difficult to implement in genetic diagnostics. Here, we show the results of our study aiming to develop a less labor-intensive (semi-automated), more high-throughput separation method to enrich intact HLA-G-positive trophoblasts from a Pap smear during pregnancies in a clinical setting. We initially tested FACS, but due to technical limitations, we continued with testing and optimizing semi-automated immunomagnetic cell sorting.

## 2. Materials and Methods

### 2.1. Inclusion Criteria for Patients and Ethical Considerations

We included pregnant women 18 years and older with a gestational age of between 6 and 20 weeks in this study. All had an indication for CVS and had provided written informed consent, including publication of the study results, during their inclusion in this study. Patients were excluded when there were fewer than 24 h between the time they received the study information and CVS sampling. This study was conducted according to the principles of the Declaration of Helsinki (Fortaleza, Brazil, October 2013 version) and in accordance with the Medical Research Involving Human Subject Act (WMO). The medical ethics committee of the University Medical Center Groningen (UMCG) approved the study protocol (NL 79181.042.22).

### 2.2. Study Design

This study was divided into two phases. Phase 1 was a pilot test simulating the enrichment of fetal HLA-G-positive cells from HLA-G-negative maternal cells using the Jeg-3 (HLA-G positive) and HeLa (HLA-G negative) cell lines using Fluorescent Activated Cell Sorting (FACS). In Phase 2, we tested the enrichment of HLA-G-positive trophoblasts from maternal cervical cells harvested from Pap smears. However, we discovered that FACS with a 100 µm nozzle was inefficient due to the size of the trophoblasts (92% of primary trophoblasts were 30–90 μm in culture [18]), and it was difficult to optimize the process with a larger nozzle. We therefore tested immunomagnetic cell sorting with cell lines like in phase 1 followed by processing of the Pap smears.

### 2.3. Culturing and Harvesting of Cell Lines

Jeg-3 (HTB-36^TM^, ATCC, Manassas, VA, USA) and HeLa (CCL-2™, ATCC) cell lines were cultured in Dulbecco’s Modified Eagle Medium (1× DMEM, Gibco, ThermoFisher Scientific, Waltham, MA, USA) + 10% Fetal Bovine Serum (Gibco, ThermoFisher Scientific) + 1% penicillin/streptomycin (Capricorn scientific, Ebsdorfergrund, Germany) in T75 culture flasks. The cultures were passed twice a week (Jeg-3 1:5 and HeLa 1:20), and leftover cells were centrifuged (10 min, 400× *g*, 37 °C), mixed in 1 mL of phosphate-buffered saline (PBS) with a needle and syringe, and stored in 20 mL of Cytolyt solution (Hologic Inc., Marlborough, MA, USA).

The Cytolyt suspension was centrifuged (10 min, 400× *g*, 4 °C) and re-suspended in 12–13 mL of ice-cold Dulbecco’s PBS (Gibco) with 0.1% BSA stock solution (Miltenyi Biotec, Bergisch Gladbach, Germany) and 2 mM EDTA. The cells were then washed twice with 12─13 mL of ice-cold PBS (centrifuge steps 10 min, 400× *g*, 4 °C). After the final wash, the cells were resuspended in PBS with 1% BSA to a concentration of 1 × 10^7^ cells/mL. Afterwards, the Jeg-3 and HeLa cells were mixed in the desired ratios (10%, 1%, 0.1%).

### 2.4. Endocervical Sampling

Endocervical sampling was performed directly after inserting the speculum and before disinfection and collection of the CVS sample, which is mainly performed transcervically at the UMCG. Sampling was performed using a standard nylon cytobrush (CooperSurgical, Inc., Trumbull, CT, USA) introduced approximately 2 cm into the endocervical canal and rotated 360° as it was withdrawn. To increase the yield of number of cells, a second cytobrush was used in the final 19 of 26 patients included. The collected cells were immediately fixed using 20 mL of Cytolyt solution (Hologic Inc.) and transported to the laboratory. The samples were stored at 4 °C until further processing.

The Cytolyt suspension was centrifuged (10 min, 400× *g*, 4 °C) and re-suspended in 12–13 mL of ice-cold PBS (Gibco) with 5% MACS BSA (Miltenyi Biotec) and 10 mM EDTA. The cells were then washed twice with ice-cold PBS with 5% MACS BSA (centrifuge steps 10 min, 400× *g*, 4 °C). After the final wash, the cells were resuspended in PBS with 5% BSA.

### 2.5. Antibody Titration and Staining for FACS

Based on a titration experiment with anti-HLA-G-PE antibody (HLA-G monoclonal antibody (MEM-G/9), MA1-19643, PE, ThermoFisher Scientific), HeLa, and Jeg-3 cell lines, we determined a concentration of 0.25 µg/100 µL to be optimal. This concentration was used for all further experiments with cell lines and endocervical samples. In each experiment, we used part of the sample as a negative control sample (no antibody). The samples were incubated for at least 1 h at 4 °C on a roller bank in the dark. After incubation, the cell line and endocervical samples were washed 3 times with ice-cold PBS (Gibco) with 1% MACS BSA (cell lines) or PBS (Gibco) and 5% MACS BSA (centrifuge steps 5 min, 400× *g*, 4 °C). After the final wash, the cell lines were diluted in 200 µL of PBS (Gibco) with 1% MACS BSA, and the endocervical samples were diluted in 600 µL of PBS (Gibco) with 5% MACS BSA. The final cell line suspensions were filtered using Falcon 5 mL round-bottom polystyrene tubes through a 35 µm nylon mesh filter (Corning, NY, USA). The 35 µm filters were pre-washed with 200 µL of PBS with 1% MACS BSA and post-washed with 400 µL of PBS with 1% MACS BSA. The final endocervical sample suspension was filtered through ProFlow 70 µm filters (Biorad, Hercules, CA, USA). The 70 µm filters were pre-washed with 600 µL of PBS with 5% MACS BSA and post-washed with 300 µL of PBS with 5% MACS BSA.

### 2.6. Enrichment of Fetal Cells Using Cell Lines with FACS

The labeled cells were sorted using a S3e (Biorad, 100 µm nozzle) or SH800S (Sony, Tokyo, Japan, 100 µm chip) cell sorter following the manufacturer’s instructions. The sorted cells were collected in PBS with 1% MACS BSA, centrifuged (5 min, 400× *g*, 4 °C), and re-suspended in Tissue Lysis (ATL) buffer (Qiagen, Hilden, Germany). DNA was extracted using the QIAamp DNA microkit (ID, Cat No. 56304) following an alternative protocol (QA43-Jul-10) with the exclusion of carrier RNA to prevent disturbances of downstream processes. The DNA was eluted in 28 µL of Tris EDTA (10 mM Tris, pH 8.0, 1mM EDTA). Quantitative fluorescence polymerase chain reaction (QF-PCR) (Devyser Complete v2, Hägersten, Sweden) was used for a quality check.

### 2.7. Enrichment of Fetal Cells from Cell Line Mixtures and Endocervical Samples Using Immunomagnetic Cell Sorting

In short, 300 µL of the 800 µL of HLA-G-PE labeled or unlabeled cell line suspensions (end-product antibody titration and staining) were diluted with 700 µL of 1% MACS BSA. The diluted cell line suspension was incubated for 30 min at 4 °C with 30 µL of anti-PE magnetic beads (Applied Cells, Santa Clara, CA, USA) on a roller bank in the dark. We tested incubation with 10, 20, 30, and 60 µL of anti-PE magnetic beads (Applied Cells). As 30 µL led to best performance, this volume was used in further experiments with cell lines and endocervical samples. After incubation, the cells were sorted using immunomagnetic cell sorting (MARS-technology, Applied Cells) following the manufacturer’s instructions (3.1, positive selection, standard flow rate). HLA-G-PE-negative cells were enriched in the negative fraction; HLA-G-PE-positive cells were enriched in the positive fraction. Re-sorting was tested in combination with 30 and 60 µL of anti-PE magnetic beads (Applied Cells) using the 1:1 mixed positive fraction as the input sample. The input sample, positive fraction, and negative fraction were analyzed using a NovoCyte Quanteon Flow Cytometer (Quanteon, Agilent, Santa Clara, CA, USA).

For the endocervical samples, 1 mL of the 1.5 mL HLA-G-PE labeled or unlabeled endocervical samples were incubated for 30 min at 4 °C with 30 µL of anti-PE magnetic beads (Applied Cells) on a roller bank in the dark. After incubation, the samples were diluted with 9 mL of PBS with 5% MACS BSA and sorted using immunomagnetic cell sorting (MARS-technology, Applied Cells) following the manufacturer’s instructions (3.1, positive selection, standard flow rate). HLA-G-PE-negative cells were enriched in the negative fraction; HLA-G-PE-positive cells were enriched in the positive fraction. The input sample, positive fraction, and negative fraction of each sample were analyzed using a NovoCyte Quanteon Flow Cytometer. Sub-samples of the positive and the negative fractions were fixed with acetic acid–methanol (1:3, 3× wash (10 min, 250 rcf)) and deposited on slides. Select slides were stained for 5 min at room temperature with Giemsa (Merck, Darmstadt, Germany) and viewed with a microscope. The other slides were labeled overnight at 37 °C with fluorescent XA X/Y Aneusomy probe mix (D-5608-100-OG, Metasystems, Heidelberg, Germany) after 2 min of denaturation at 72 °C. Successively, the slides were washed with 0.4× saline–sodium citrate (SSC) and 2 × SSC/0.5%Tween, and at least 500 nuclei were analyzed using a fluorescence microscope.

## 3. Results

### 3.1. Enrichment of Fetal Cells from Cell Line Mixtures with FACS

**Figure 1:**
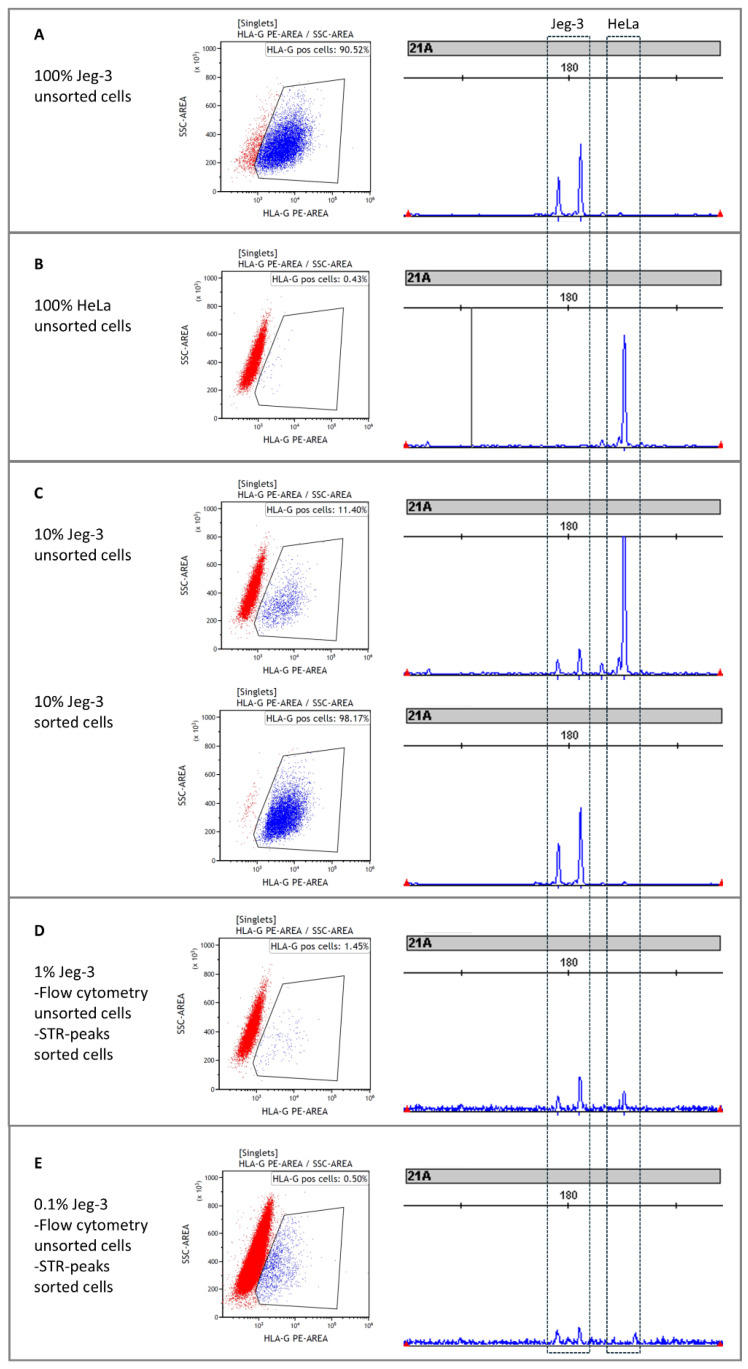
FACS sorting of HLA-G-positive Jeg-3 cells from a background of HLA-G-negative HeLa cells. On the left of each panel, flow cytometry plots are shown of different dilutions of Jeg-3 cells in an environment of HeLa cells with the gates set for HLA-G-PE-positive cells. On the right of each panel, STR peaks are depicted of the cells in the sorted fractions. The lengths of the Jeg-3 and HeLa-specific STR marks are indicated with a box. (**A**) FACS plot and STR lengths of 100% JEG3 cells; (**B**) FACS plot and STR lengths of 100% HeLa cells; (**C**) FACS plots of unsorted and sorted mixture of 10%JEG3/90%HeLa cells and STR lengths detected of both cell types; (**D**) FACS plot of unsorted mixture of 1% JEG3 cells/99% HeLa cells and JEG3 and HeLa STR lengths observed in the sorted sample; (**E**) FACS plot of unsorted mixture of 0.1% JEG3 cells/99.9% HeLa cells and JEG3 and HeLa STR lengths observed in the sorted sample.

A dilution series of 10%, 1%, and 0.1% stained HLA-G-positive Jeg-3 on a background of HLA-G-negative HeLa cells showed expected percentages after sorting with FACS (Figure 1). HLA-G-positive cells were strongly enriched in the 10% Jeg-3 sorted sample (Figure 1). The other sorted samples were not re-analyzed with FACS because of the limited number of cells. Enrichment of Jeg-3 cells was confirmed with QF-PCR, where Jeg-3-specific short tandem repeats (STR) were strongly enriched in the sorted samples compared to the input samples (Figure 1). We repeated this experiment twice, with comparable results.

### 3.2. Enrichment of Fetal Cells from Cell Lines with Immunomagnetic Cell Sorting

**Figure 2:**
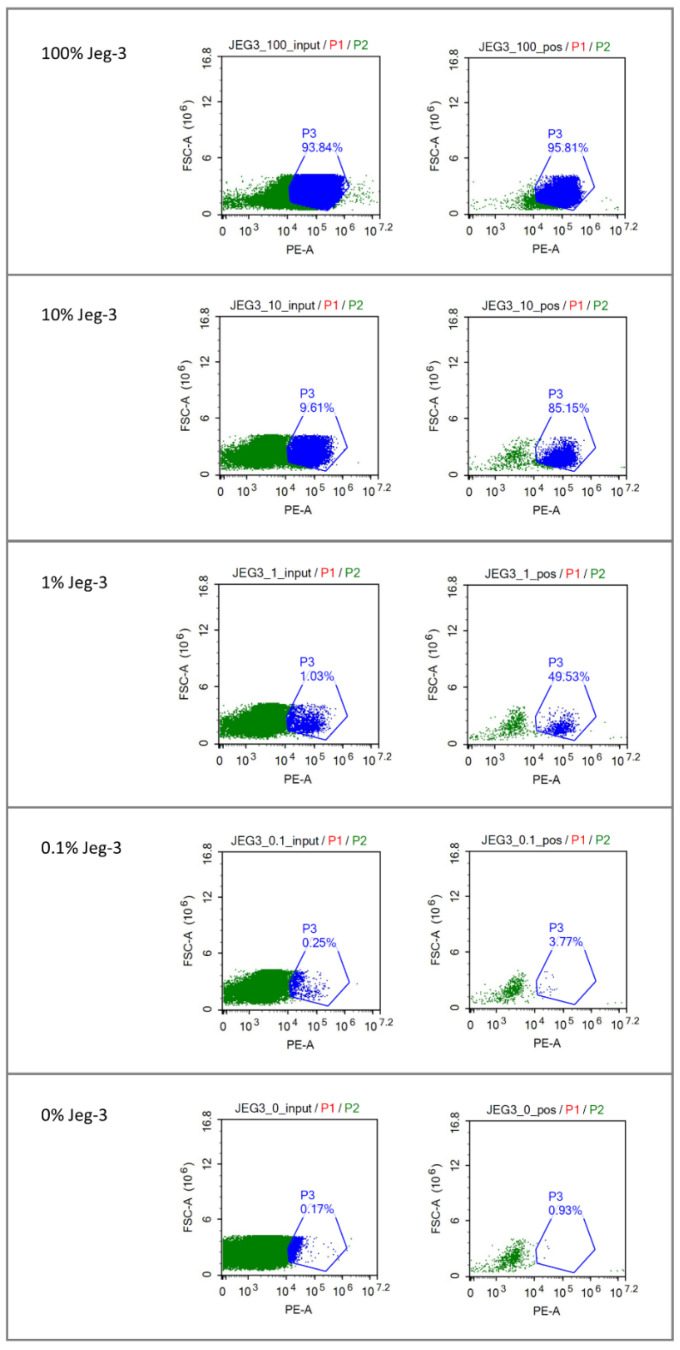
Immunomagnetic cell sorting of HLA-G-positive Jeg-3 cells from a background of HLA-G-negative HeLa cells. Flow cytometry images are shown of different dilutions of Jeg-3 cells (100%, 10%, 1%, and 0.1%) in an environment of HeLa cells with the FACS gates set for HLA-G PE-labeled positive cells. On the left of each panel, the percentage of Jeg-3 cells in the input sample (JEG3_100_input, JEG3_10_input, JEG3_1_input, JEG3_0.1_input, JEG3_0_input) is shown; on the right of each panel, the corresponding HLA-G PE-labeled positive fractions after immunomagnetic cell sorting (JEG3_100_pos, JEG3_10_pos, JEG3_1_pos, JEG3_0.1_pos, JEG3_0_pos) are depicted.

Immunomagnetic cell sorting of the stained HLA-G-positive Jeg-3 cells against a background of HLA-G-negative HeLa cells resulted in substantial enrichment of Jeg-3 cells. In four independent dilution series, the mean percentages of Jeg-3 cells went from 7.1% to 53.5% (7.5-fold enrichment), 0.9% to 32.6% (36-fold enrichment), and 0.4% to 2.6% (6.5-fold enrichment). The results of a representative experiment are shown in Figure 2. We tested whether increasing the anti-PE magnetic bead concentration leads to higher enrichment, but this had a negative effect. Re-sorting the positive fractions had a limited positive effect on enrichment but a negative effect on the total number of cells in the positive fractions. This was therefore not implemented.

### 3.3. Enrichment of Fetal Cells from Endocervical Samples

We included 26 pregnant women in this study, with a mean age of 31.3 (range: 25–38 years) and a mean gestational age of 12 weeks and 3 days (Table 1). Initially, we collected one cytobrush per patient. However, we only detected a visible cell pellet in three of the first seven samples (Table 1). We therefore decided to collect two brushes for subsequent patients. Even when using two brushes, we only collected visible cell pellets in 10 of the next 19 samples (Table 1). Despite this moderate yield, we tried immune-magnetic fetal cell enrichment from the endocervical samples with sufficient cells.

**Figure 3:**
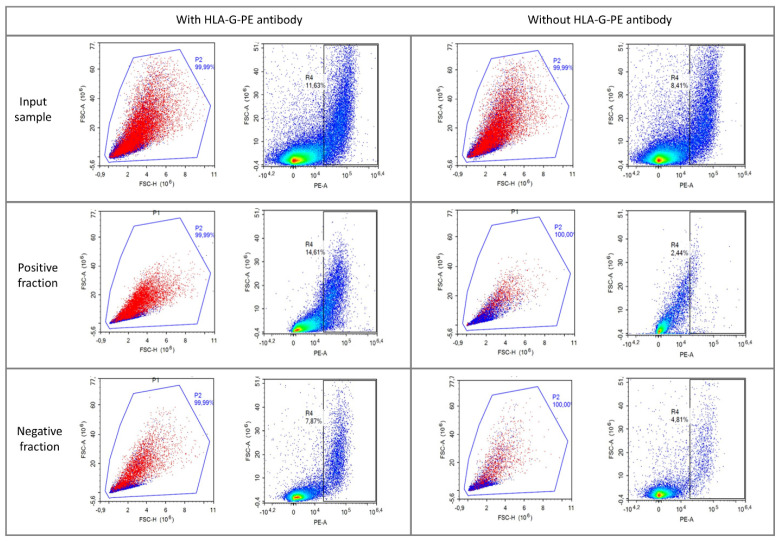
Representative flow cytometry results of the input sample before (top row) and after immunomagnetic sorting (2nd and 3rd rows). The HLA-G- positive fraction (2nd row) and the HLA-G- negative fraction (3rd row) of a stained (left) and unstained (right) endocervical sample of the same patient show enrichment of HLA-G- positive cells in the sorted fraction of the stained sample compared to the unstained sample. Epithelial and potential fetal trophoblast cells were gated (indicated with p2), and an HLA-G-PE-positive threshold was set based on the unstained sample.

A cervical swab consists of large maternal epithelial cells, large fetal trophoblasts (92% of primary trophoblasts are 30–90 μm in culture [18]), and small maternal endocervical cells. After immunomagnetic cell sorting, HLA-G-PE-negative cells should be enriched in the negative fraction, and HLA-G-PE-positive cells should be enriched in the positive fraction. Flow cytometry was used for confirmatory analysis of the input sample and both fractions. Maternal epithelial cells and fetal trophoblasts were distinguished from maternal cervical cells based on cell size (gate P2, Figure 3), and epithelial cells and trophoblasts were further distinguished based on the threshold for HLA-G-PE intensity (Figure 3). The much smaller maternal cervical cells were left ungated because they are not distinguishable from cell debris with scatter parameter settings, but they are present in the samples. Using these settings, the HLA-G-positive cells were moderately increased in the positive fraction (14.61 vs. 11.63%) and decreased in the negative fraction (7.87 vs. 11.63%) compared to baseline pre-sorting (Figure 3). Enrichment of positive cells was not shown in the unstained sample (Figure 3).

**Figure 4:**
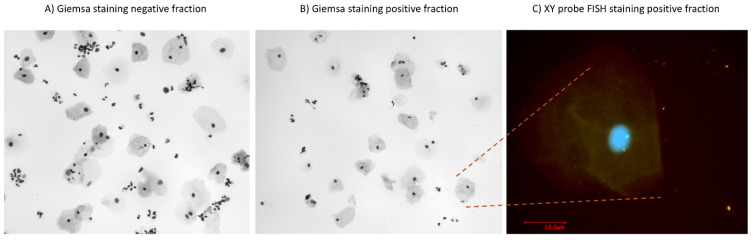
Giemsa staining of the negative and positive fractions after immunomagnetic cell sorting and fluorescent in situ hybridization for XY chromosomes. (**A**) Endocervical cells (nuclei with less cytoplasm) are enriched in the Giemsa-stained negative fraction. (**B**) Epithelial and fetal trophoblast cells (nuclei with more cytoplasm) of a male fetus are enriched in the Giemsa-stained positive fraction. Epithelial and fetal trophoblasts cannot be distinguished based on Giemsa staining. (**C**) Fluorescent in situ hybridization shows two green fluorescent labels in each nucleus, representing two maternal X-chromosomes. At least 500 nuclei were analyzed using a fluorescence microscope.

We used Giemsa staining as a post-sorting control on the positive and negative fractions and observed more large cells of potential fetal origin in the positive fraction, while small cervical cells were enriched in the negative fraction (Figure 4). We used a sample from a mother pregnant with a male fetus to check whether the large cells were of fetal origin using fluorescent in situ hybridization (FISH) with XY probes. However, all of the cells were maternal, as two green fluorescent labels were visible per nucleus, representing two X-chromosomes, and no male signatures were detected in the sample (Figure 4).

## 4. Discussion

We were able to enrich an HLA-G-positive Jeg-3 cell line against a background of HLA-G-negative HeLa cells using FACS and semi-automated immunomagnetic cell sorting. Enrichment of HLA-G-positive trophoblasts from an endocervical sample is inefficient with FACS because of the diameter of the cells [18], but enrichment should be possible with immunomagnetic cell sorting. We enriched HLA-G-PE antibody-stained cells from an endocervical sample from a woman with a male fetus (Figure 3). FISH staining for XY chromosomes in the positive immunomagnetic cell sorting fraction showed only XX signatures and no male signature despite visual inspection of at least 500 nuclei, which should be sufficient to detect at least one XY nucleus (Figure 4). A low percentage of the maternal epithelial samples were a-specifically stained and thus sorted in the positive fraction. We therefore suspect that no fetal cells—or only a negligible number—were present in the endocervical swabs.

We could only collect sufficient numbers of cells for further processing from ~50% of the swabs despite using two swabs per collection. Although more successful, a previous study by van Dijk et al. also found that multiple samples had cell numbers that were too low for further processing or had the maternal phenotype only [17]. Using the TRIC method [15], they were able to acquire a fetal fraction of >20% in 44% of the cases, and they were able to identify SNVs carried by the fetus in 23% of the cases. A similar enrichment of fetal cells from a swab was shown by Lee et al. [19]. In another study, Bourlard et al. tried, as we did, to enrich fetal cells from endocervical samples with FACS and immunomagnetic cell sorting [20], but they were not able to enrich HLA-G-positive cells with FACS, possibly due to the diameter of the cells of interest. Furthermore, digital droplet PCR for Y chromosomal markers were unreliable. Even though the semi-automated immune magnetic cell sorting we used is not hampered by cell size, we were, unfortunately, also not able to detect fetal cells. Notably, these studies [17,19,20] and our study were performed in a clinical setting with ongoing pregnancies, which is in contrast to the original study describing the TRIC method, which used samples from terminated pregnancies [15]. In our study, endocervical sampling of 26 ongoing pregnancies was performed. The very careful sampling in this setting likely limited the harvest of fetal cells in comparison to the original TRIC study. Our study reinforces the conclusion of previous studies that endocervical sampling of fetal cells is difficult to implement in a clinical setting of ongoing pregnancies [17,20]. Even when endocervical sampling is developed into a successful method for genetic testing, it will be necessary to confirm genetic aberrations using amniocentesis to exclude false-positive results caused by mosaicism resulting from the origin of the cells [21].

Ultimately, intact fetal cells will be needed to detect single-gene defects in a cost-effective manner in a clinical setting. Although our current results on clinical samples are modest, we think that immunomagnetic cell sorting could be a non-labor-intensive, semi-automated method to enrich fetal cells with a unique cell marker. However, for this to work, we will need to find a source of sufficient fetal cells that is easier to sample. If the fetal enrichment after immunomagnetic cell sorting is insufficient for direct-targeted or whole-genome sequencing, there are options to further increase sequencing yield from fetal cells; for example, via shallow sequencing of dispensed pools of cells followed by deep sequencing of fetal DNA-positive pools.

## Figures and Tables

**Table 1:** Patients and samples. The characteristics of included patients, the number of brushes collected, and their yields are depicted in this table. The number of cells is defined as a usable cell pellet (+) or a hardly visible or absent cell pellet (−).

Sample Number	Age of Mother	Number of Pregnancies	Gestational Age (w;d)	Number of Brushes	Number of Cells
1	30	3	11;1	1	+
2	28	3	11;6	1	−
3	33	4	11;1	1	−
4	32	6	11;0	1	+
5	38	6	11;2	1	−
6	29	4	11;5	1	+
7	25	2	11;5	1	−
8	31	4	11;1	2	+
9	31	1	11;2	2	+
10	27	1	13;2	2	−
11	32	2	13;1	2	+
12	26	5	12;0	2	+
13	34	4	11;1	2	+
14	29	2	11;6	2	+
15	30	1	11;1	2	−
16	38	1	13;1	2	+
17	35	4	11;3	2	−
18	33	4	13;6	2	−
19	28	2	14;2	2	−
20	34	2	12;1	2	−
21	36	2	13;1	2	+
22	31	5	11;5	2	−
23	34	2	11;5	2	−
24	32	3	12;0	2	−
25	25	1	20;3	2	+
26	33	4	11;5	2	+

## Data Availability

The data that support the findings of this study are available from the corresponding author, E.N.d.B., upon reasonable request.

## Abbreviations

NIPT, non-invasive prenatal testing; cffDNA, cell-free fetal DNA; CNV, copy number variation; SV, structural variant; SNV, single-nucleotide variants; CVS, chorionic villus sampling; WES, whole-exome sequencing; Pap smear, Papanicolaou smear; TRIC, Trophoblast Retrieval and Isolation from the Cervix; FACS, Fluorescent Activated Cell Sorting; QF-PCR, quantitative fluorescence polymerase chain reaction; FISH, fluorescent in situ hybridization.
